# Research on Creeping Flashover Characteristics of Nanofluid-Impregnated Pressboard Modified Based on Fe_3_O_4_ Nanoparticles under Lightning Impulse Voltages

**DOI:** 10.3390/nano9040524

**Published:** 2019-04-03

**Authors:** Bingliang Shan, Meng Huang, Yupeng Ying, Mingkang Niu, Qian Sun, Yuzhen Lv, Chengrong Li, Bo Qi, Zhaoliang Xing

**Affiliations:** 1 State Key Laboratory of Alternate Electrical Power System with Renewable Energy Sources, North China Electric Power University, Beijing 102206, China; shanbingliang521@163.com (B.S.); 18811360731@163.com (Y.Y.); niumingkang@163.com (M.N.); lcr@ncepu.edu.cn (C.L.); lqicb@163.com (B.Q.); 2 School of Energy, Power and Mechanical Engineering, North China Electric Power University, Beijing 102206, China; 15652912576@163.com (Q.S.); yuzhenlv@163.com (Y.L.); 3 State Key Laboratory of Advanced Power Transmission Technology, Global Energy Interconnection Research Institute Co. Ltd., Beijing 102209, China; 15811444029@163.com

**Keywords:** Fe_3_O_4_ nanoparticles, creeping flashover, nanofluid-impregnated pressboard, shallow traps

## Abstract

Creeping flashover of mineral-oil-impregnated pressboard under impulse stress is a common insulating failure in oil-immersed transformers, arousing increasing attention. Recent studies have shown that the breakdown strength of transformer oil under positive lightning impulse voltage can be significantly improved through nanoparticles-based modification, and Fe_3_O_4_ has shown the best improvement in breakdown strength compared to other nanoparticles that have been used. This paper presents the creeping flashover characteristics of pure oil-impregnated pressboard (OIP) and nanofluid-impregnated pressboard (NIP) based on Fe_3_O_4_ nanoparticles under positive and negative lightning impulse voltages, respectively. It was found that NIP possessed higher resistance to creeping flashover than OIP. The relative permittivities of oil and oil-impregnated pressboard before and after nanoparticles-based modification were measured, and the results revealed that the addition of nanoparticles led to a better match in relative permittivity between oil and oil-impregnated pressboard, and a more uniform electric field distribution. Furthermore, the shallow trap density in NIP was obviously increased compared to that of OIP through the thermally stimulated depolarization current (TSDC), which promoted the dissipation of surface charges and weakened the distortion of the electric field. Therefore, the creeping flashover characteristics of oil-impregnated pressboard were greatly improved with Fe_3_O_4_ nanoparticles.

## 1. Introduction

Oil/pressboard composite insulation systems have been widely applied in large power transformers due to their excellent dielectric properties and relatively high mechanical strength [1]. The interface between oil and pressboard is usually regarded as the insulating weak point, where surface charges easily accumulate under external stress, leading to distortion of the local electric field and insulation failure [2,3]. Significant research efforts have been devoted to the creeping flashover characteristics of oil-impregnated pressboard (OIP), and theoretical research on the mechanism process has seen rapid growth in the past decades [4,5]. The previous literature has demonstrated that the generation and development of creeping flashover is closely related to local electric stress, dielectric strength, and moisture content of the insulating materials [4,5,6,7]. Thus, effective measures must be taken to weaken the distortion of local electric fields and improve the dielectric performance of materials so as to restrain the occurrence of creeping flashover in oil-impregnated pressboard and assure the safe operation of transformers. 

Recent studies have shown that the dielectric performance of mineral oil can be improved through the modification of nanoparticles, which has gained enormous attention [8,9,10,11,12,13,14,15,16,17,18]. It has been illustrated that the AC breakdown strength of pure oil can be improved by the addition of Fe_3_O_4_ [11], TiO_2_ [12,13], ZnO [13], and SiO_2_ [14] nanoparticles. The analysis of Lee et al.’s study shows that the AC breakdown strength of nanofluid modified with Fe_3_O_4_ nanoparticles is two times higher than that of pure oil and that the modification effect is related to the external field, even though the dielectric loss of Fe_3_O_4_ nanofluid has been found to be larger than that of mineral oil [11]. Kopčanský et al. confirmed that the DC breakdown voltage of transformer oil could be increased by using Fe_3_O_4_ nanoparticles [15]. However, when Kudelcik et al. subjected Fe_3_O_4_ nanofluid to an external magnetic field of 20 mT, the improvement of DC breakdown strength was reduced [16]. Segal et al. found that the positive impulse breakdown voltage of nanofluid using Fe_3_O_4_ nanoparticles was increased by up to 50% when compared to that of pure oil [17]. The lightning impulse breakdown strengths of pure oil and various nanofluids have also been studied by Zhou et al. and their results showed that the insulating property of pure oil could be improved with the addition of nanoparticles [9]. They concluded that Fe_3_O_4_ nanoparticles, with unique electrical and physical properties, showed great potential in improving the dielectric performance of transformer oil compared to the potential of other nanoparticles. Meanwhile, the dielectric strength of vegetable-oil-based nanofluid could also be obviously enhanced with Fe_3_O_4_ nanoparticles [18].

Since the pioneering work of Segal et al., it was reported for the first time that Fe_3_O_4_ nanoparticles could move freely in and out of nanofluid-impregnated pressboard (NIP) without binding to cellulose [19]. This phenomenon has provided a new idea for improving the dielectric properties of OIP by immersing the pressboard in the transformer-oil-based nanofluid modified using Fe_3_O_4_ nanoparticles, since these nanoparticles would not be blocked out of the pressboard. To date, no work on the creeping flashover characteristics of NIP based on Fe_3_O_4_ nanoparticles under lightning impulse voltages has been reported, which deserves further study.

The present work aims to investigate the effects of Fe_3_O_4_ nanoparticles on the creeping flashover behaviors of oil-impregnated pressboard under lightning impulse stress. Dielectric permittivity testing and electrostatic probe techniques were used to measure both the relative permittivities and space charge behaviors of oil/pressboard before and after modification by Fe_3_O_4_ nanoparticles. Combined with the results of thermally stimulated depolarization current (TSDC), the mechanism of creeping flashover characteristics modification is proposed.

## 2. Experiment

### 2.1. Samples Preparation

**Figure 1:**
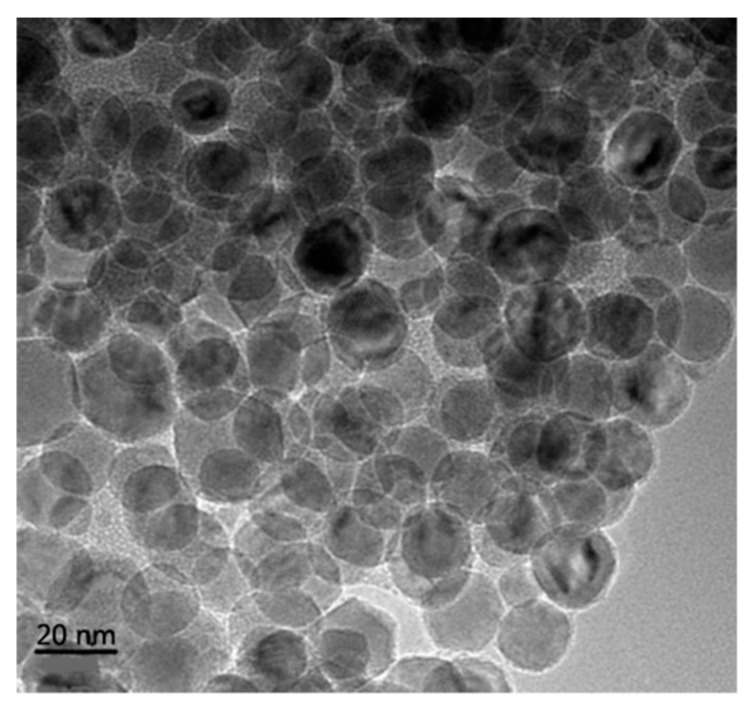
TEM of Fe_3_O_4_ nanoparticles.

The transformer oil (KI 25X) was strictly filtered so as to meet the requirement of pure oil proposed by the Conseil International des Grands Réseaux Électriques (CIGRE) working group 12.17 [20]. Fe_3_O_4_ nanoparticles were prepared by the chemical co-precipitation method using iron powder and FeCl_3_·6H_2_O as sources of iron, and modified by oleic acid to prevent the aggregation of nanoparticles. The nanoparticles obtained possessed an average diameter of 20 nm and there was no agglomeration, as shown in Figure 1. The nanofluid was prepared by evenly dispersing Fe_3_O_4_ nanoparticles into filtered oil by the ultrasonic method with a concentration of 0.2 g/L. The power of the ultrasonic dispersion device was set to 50 W/L and the ultrasonic dispersion operation lasted no less than half an hour. Pressboard samples with a size of 85 mm × 60 mm × 2 mm were dried in a ventilated oven at 105 °C for 48 h. Then, pressboard samples and oil samples were treated under vacuum below 1 kPa at 80 °C for 48 h to get rid of moisture and air, and the moisture content of the oil samples gained was 10 ppm. After that, the treated pressboards were immersed into the dry oil and nanofluid respectively, then placed under vacuum below 1 kPa for 48 h. Finally, pressboard samples with a moisture content of 0.5% were obtained.

### 2.2. Impulse Creeping Flashover Test

**Figure 2:**
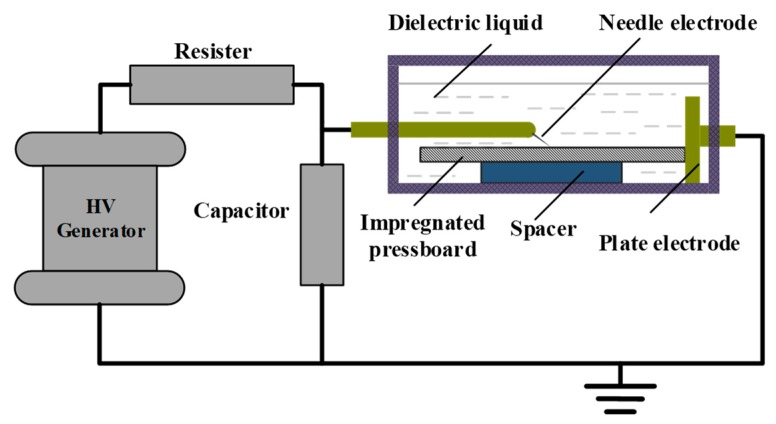
Experimental setup for creeping flashover tests.

The creeping flashover test was performed in an insulating container measuring 120 mm × 80 mm × 50 mm, as schematically illustrated in Figure 2. A needle-plane electrode system, where the steel needle (tip radius of 35 ± 5 μm) was placed at 30° to horizontal and connected to the high-voltage generator opposing a copper plate electrode connected to the ground, was located in the container. The oil-impregnated pressboard was placed horizontally in tight contact with two electrodes and the distance between needle tip and the plane could be adjusted from 20 to 40 mm. In addition, pure oil was selected as the dielectric liquid for the OIP test and nanofluid as the dielectric liquid for the NIP test.

During the test, an impulse voltage generator was used to provide 1.2/50 μs standard lightning impulse waveforms. For the validity of the test, the step-by-step method was used in the testing process. At the beginning of tests, a selected proper value of impulse voltage was applied 3 times. If the creeping flashover did not happen, the voltage was increased by 5 kV and applied to the samples another three times. The previous steps were repeated until creeping flashover occurred. The time interval between experiments was at least 2 min, and six effective creeping flashover trials were successfully conducted.

### 2.3. Relative Permittivity Test

The relative permittivities of oil samples and pressboard samples were tested using a Jiantong dielectric impedance instrument (Baoding, China) according to IEC 60250 standards and Novocontrol Concept 40 broadband dielectric spectrometer (Montabaur, Germany), respectively. Each sample was tested five times and then the average value was recorded.

### 2.4. Measurement of Trap Distribution Characteristics

**Figure 3:**
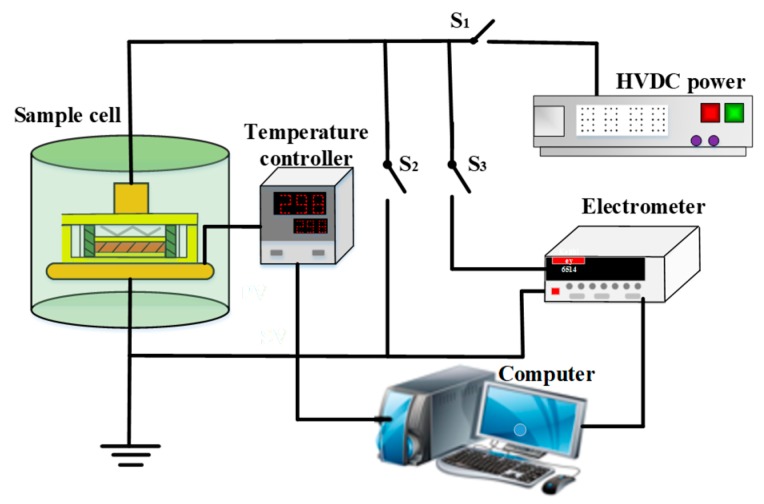
Schematic of thermally stimulated depolarization current (TSDC) measurement.

Trap distribution characteristics of NIP and OIP were measured through TSDC, with the schematic diagram is shown in Figure 3. First, the test sample was heated to 313 K before being polarized under a negative DC voltage of 4 kV for 30 min. Secondly, the temperature of the sample was quickly lowered to 263 K and then the polarization voltage was cut off. After that, the two sides of the sample were shorted for 2 min in order to release the free charges. Finally, the sample was heated at a rate of 2 K/min until it reached 353 K when the depolarization current was simultaneously measured by a Keithley 6514 electrometer (Tektronix Inc., Beaverton, OR, USA).

### 2.5. Measurement of Surface Charge Density

**Figure 4:**
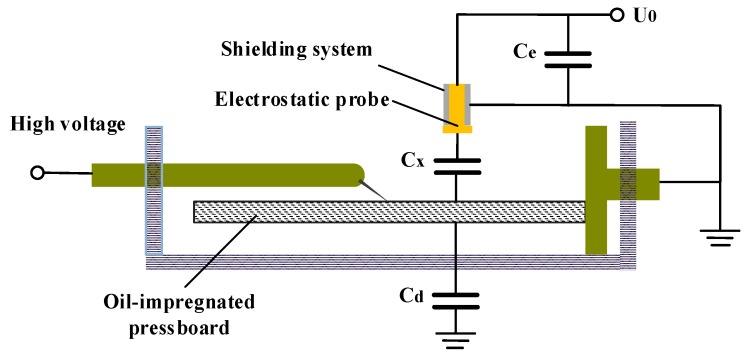
Schematic of TSDC measurement.

The surface charge densities of OIP and NIP were measured by an electrostatic probe and the diagram is schematically illustrated in Figure 4, where the gap distance between needle tip and plate electrode was set to 30 mm. The test system was based on the theory of capacitive probe, which has been widely used in research on the deposited charge at the surface of insulators. The polarization voltage of –10 kV was applied to the needle electrode for 30 min to generate space charges, and the electrostatic probe was used to measure the surface charge accumulation characteristics of samples at 5 min intervals. Power was then shut off and the decay features of the surface charges were measured as well.

## 3. Results and Discussion

### 3.1. Lightning Impulse Creeping Flashover Characteristics

**Figure 5:**
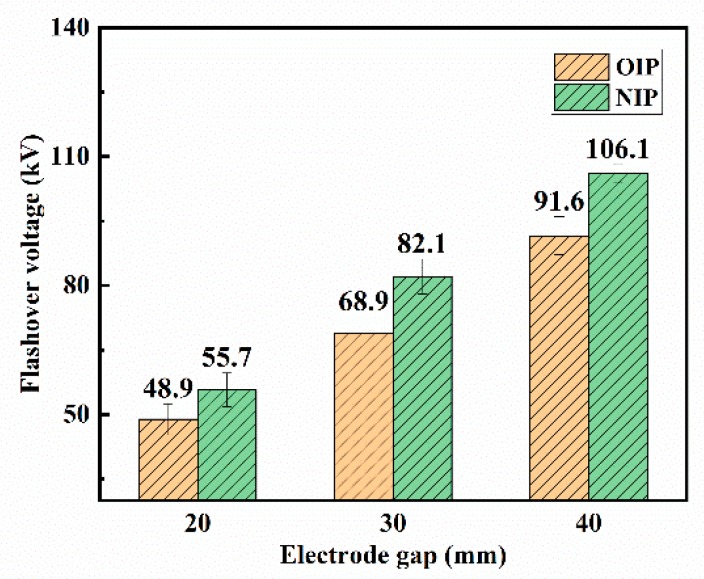
Creeping flashover voltage of the oil-impregnated pressboard (OIP) and the nanofluid-impregnated pressboard (NIP) under positive lightning impulse voltages.

The creeping flashover voltages of OIP and NIP under positive lightning impulse voltages are recorded in Figure 5. It appeared that the average creeping flashover voltages of these two samples increased with the expansion of the electrode gap distance. When compared, the average flashover voltage of NIP was apparently higher than that of the OIP under various electrode gap distances. Moreover, the improvement of average creeping flashover voltage of OIP could be increased to 1.19 times when modified with Fe_3_O_4_ nanoparticles and the electrode gap distance was set to 30 mm.

**Figure 6:**
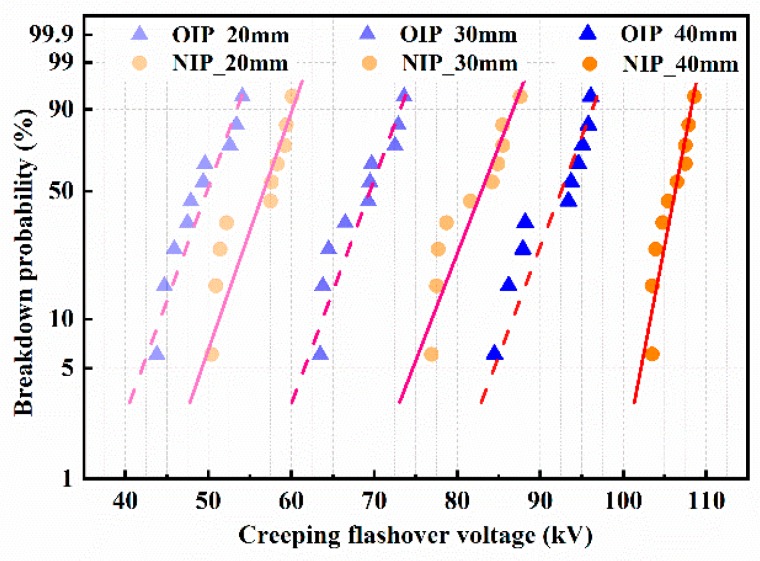
The Weibull distribution fitting curves of the creeping flashover voltages under positive lightning impulse voltages.

The Weibull distribution is commonly used for analyzing the breakdown data of electrical insulation [21]. The Weibull distribution fitting curves of the creeping flashover voltages of pressboard samples under positive lightning impulse voltages were calculated and are presented in Figure 6. Here, it is visually presented that the breakdown probability of OIP and NIP increased with the applied voltages under various electrode gap distances. Meanwhile, NIP possessed a lower breakdown probability compared to OIP when the same voltages were applied under various electrode gap distances, which is beneficial in improving the dielectric performance of materials.

The average value (AVG) and SD of chop time for samples are shown in Table 1. It is of great significance to note that the average chop time of oil-impregnated pressboard could be apparently prolonged by the addition of nanoparticles. For example, the chop time of oil-impregnated pressboard under positive lightning impulse voltages was prolonged by 72.8% when the electrode gap distance was 20 mm, and the improvement in chop time was still relatively large when the electrode gap distance was increased to 30 or 40 mm. These results are in accordance with our previous work on the streamer characteristics of Fe_3_O_4_ nanofluid [18,22], indicating that Fe_3_O_4_ nanoparticles can effectively restrain the process of creeping flashover at the oil/pressboard interface, thus extending the development time and reducing the average propagation speed of the positive streamer.

**Figure 7:**
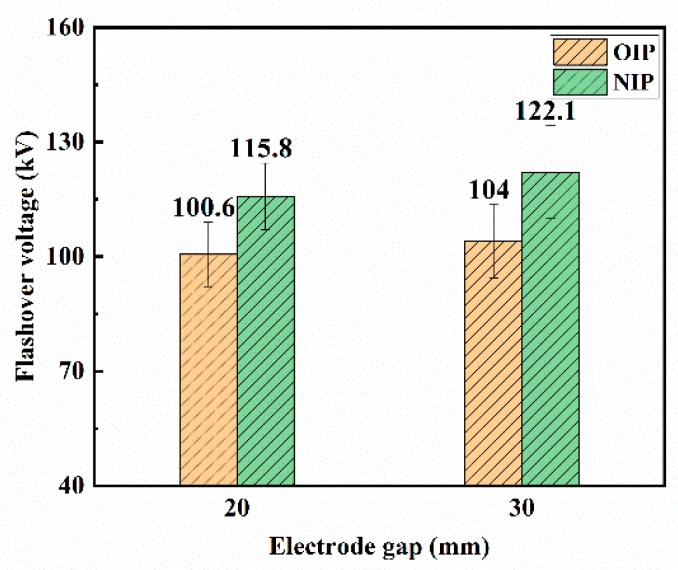
Creeping flashover voltages of OIP and NIP under negative lightning impulse voltages.

**Figure 8:**
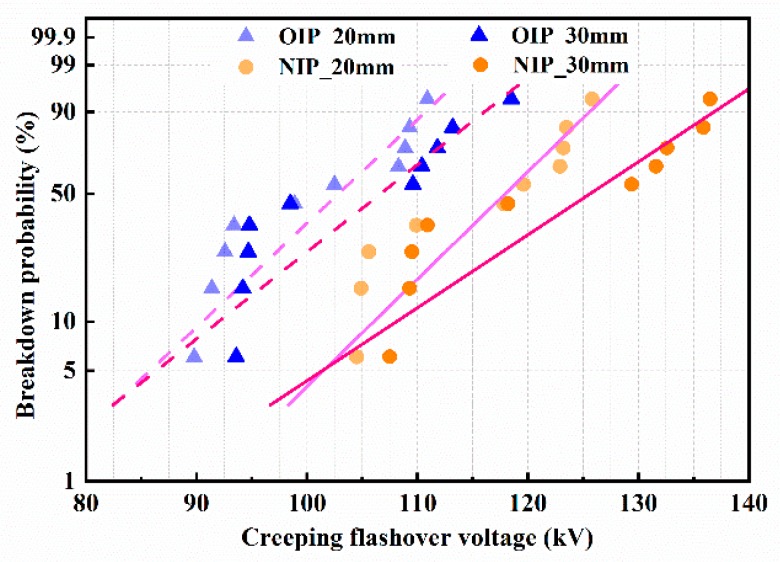
The Weibull distribution fitting curves of the creeping flashover voltages under negative lightning impulse voltages.

Since the flashover always occurred along the surface of the insulating container instead of along the pressboard samples under negative lightning impulse voltages, owing to the insufficient insulation distance between the container and the electrodes when the electrode gap distance was 40 mm, the negative flashover measures were carried out under the electrode gap distance of 20 mm and 30 mm. The creeping flashover voltages and the corresponding Weibull distribution fitting curves of OIP and NIP under positive lightning impulse voltages are shown in Figure 7 and Figure 8, respectively.

The average creeping flashover voltage increased with the expansion of the electrode gap distance under applied voltages, which is similar to the results under positive lightning impulse voltages. The creeping flashover performance of OIP was still apparently improved by modification with Fe_3_O_4_ nanoparticles. Nevertheless, it is worth noting that the creeping flashover voltage of OIP under relatively low breakdown probabilities (e.g., 5%) was almost the same or a little higher than that of NIP owing to the wider dispersion of the breakdown voltages of NIP.

The average chop time of OIP and NIP under negative lightning impulse voltage is demonstrated in Table 2. It can be seen that the differences in chop time under the same electrode gap distance was relatively small, distinct from the results under positive lightning impulse voltage. These differences may arise from the different development processes of positive streamers and negative streamers [10].

From the experimental results above, it can be reasonably concluded that NIP possessed higher resistance to creeping flashover than OIP under lightning impulse voltages. Simultaneously, the average chop time under positive lightning impulse voltages could also be prolonged, which is of great significance for restraining the propagation of the positive streamer along the surface and for enhancing the dielectric performance of oil/pressboard composite insulation systems in transformers.

### 3.2. Effect of Change in Relative Permittivities

The creeping discharge at the interface between oil and pressboard is tightly related to the electric field distribution, wherein the stronger the local tangential electric field at the interface, the more easily the creeping flashover occurs [23]. Since the lightning impulse voltage is mainly composed of high-frequency alternating components [24], the process of analyzing electric field distribution along the interface between oil and pressboard is similar to that under AC stress.

**Figure 9:**
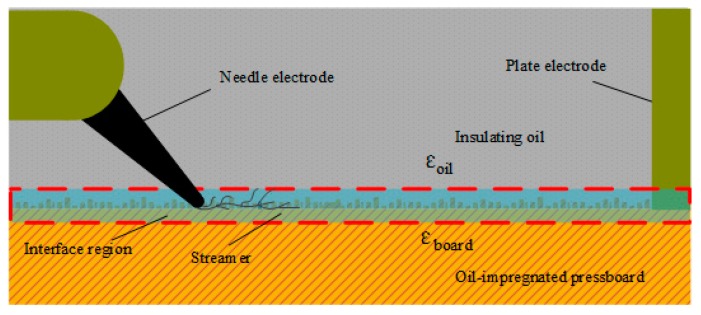
The structure schematic of the oil/pressboard interface.

The structure of the oil/pressboard interface is shown in Figure 9. Owing to the production process of pressboard, there were many grooves on the surface of the pressboard filled with insulating oil [25]. The tangential electric field distribution was calculated and analyzed from current density as follows [26]:

(1) $$J=\sigma E+\frac{dD}{dt}=\sigma E+\varepsilon_{0}\varepsilon_{r}\frac{dE}{dt}=E\left(\sigma +\varepsilon_{0}\varepsilon_{r}\omega \right),$$

where J is the tangential current density, σ is conductivity, E is the tangential electric field strength, D is electric displacement vector, $\varepsilon_{0}=8.85\times 10^{-12}$ F/m is vacuum permittivity, $\varepsilon_{r}$ is relative permittivity, and ω is the angular frequency.

The typical conductivity of insulating pressboard is about 10^−15^ S/m and the conductivity of pure oil is approximately 10^−12^ S/m, while the conductivity of nanofluid based on Fe_3_O_4_ nanoparticles is almost 10–500 times that of pure oil [26]. From Equation (1) and the parameters mentioned above, it can be deduced that $\sigma \ll \varepsilon_{0}\varepsilon_{r}\omega$ under an AC electric field. In other words, the tangential electric field distribution is mainly inversely proportional to the relative permittivity in this condition.

The relative permittivities of oil and oil-impregnated pressboard were measured, as shown in Table 3. Since the relative permittivity of pure oil is almost half that of OIP, the electric field strength in oil would be higher under applied lightning impulse voltages. Meanwhile, it has been reported the breakdown strength of OIP is usually greater than that of pure oil [23,25] and as a result, creeping flashover usually starts from pure oil. With the addition of Fe_3_O_4_ nanoparticles, the relative permittivity of pure oil-based nanofluid was enhanced from 2.2 to 3.0, while that of NIP increased by just 0.2. The improvement in relative permittivity was probably due to the additional polarization generated by Fe_3_O_4_ nanoparticles [27].

For a deeper analysis of the modification effect, based on the change in relative permittivities, on creeping discharge in the nanofluid/pressboard insulation system, the permittivity ratio of oil to oil-impregnated pressboard ($\varepsilon_{oil}/\varepsilon_{board}$) was also defined and used to measure the electric field distribution along the oil/pressboard interface region, as shown in Table 3. A better match in relative permittivity between pressboard and nanofluid was observed since the permittivity ratio was increased from 0.52 to 0.68, which helped to reduce the electric field strength in oil and enhanced that in NIP instead, leading to a more uniform electric field distribution at the interface and preventing the occurrence of creeping flashover. Moreover, previous literature has proven that the dielectric strength of Fe_3_O_4_ nanofluid is much higher than that of mineral oil [15,17,28], which also contributed to improving the dielectric performance of nanofluid/pressboard composite insulation systems. Thus, the creeping flashover voltage of oil-impregnated pressboard can apparently be improved due to the higher dielectric strength of Fe_3_O_4_ nanofluid and the more reasonable electric field distribution.

### 3.3. Effect of Shallow Trap Characteristics

**Figure 10:**
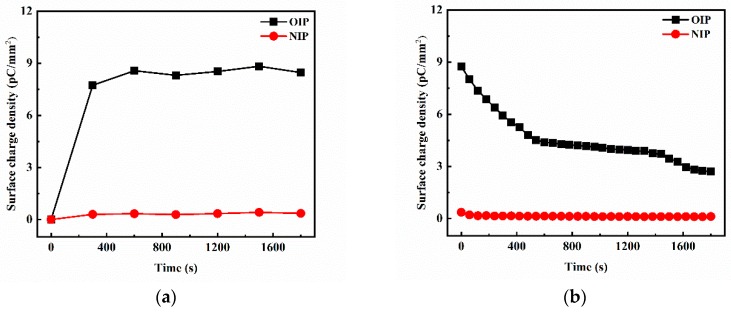
(**a**) Charge accumulation characteristics and (**b**) charge dissipation characteristics of OIP and NIP samples.

It can be deduced that the creeping flashover characteristics of oil-impregnated pressboard can also be affected by the transport characteristics of charge carriers based on the creeping discharge mechanisms of a dielectric solid [29]. Therefore, the surface charge accumulation and dissipation characteristics of OIP and NIP were tested, as shown in Figure 10. 

From Figure 10a, it can be seen that the surface charge density of OIP reached a saturated state after applying a voltage for about 600 s while that of NIP increased for 300 s before reaching a steady value. The maximum surface charge density of OIP (about 8.54 pC/mm^2^) was considerably higher than that of NIP (about 0.34 pC/mm^2^) under the same applied voltage, indicating that more space charge carriers had accumulated on the surface of the OIP. While there was a continuous decline of charge carriers on the surface of OIP after the external electric field was removed, the surface charge density was still high (about 2.71 pC/mm^2^) after 30 min. On the contrary, NIP possessed a rapid surface charge decay rate, which was beneficial to effectively prevent surface charge accumulation.

**Figure 11:**
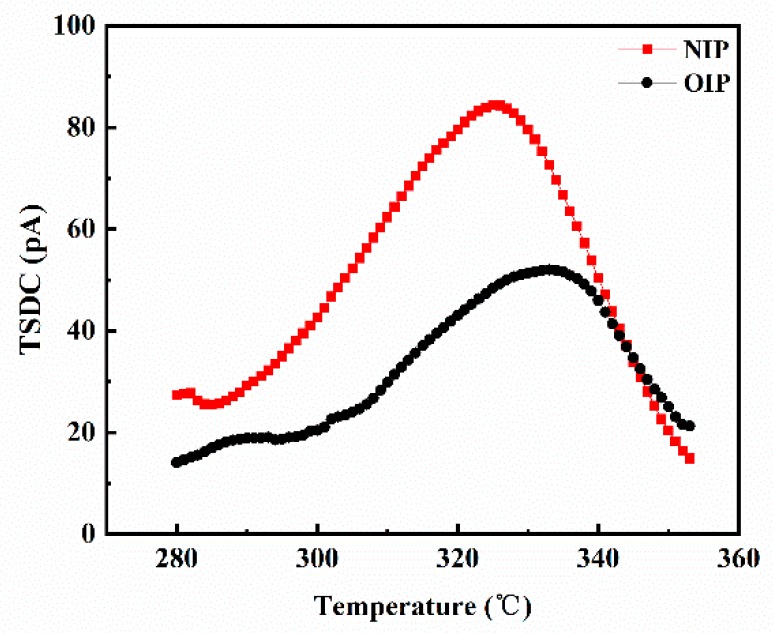
The test curves of TSDC for OIP and NIP.

It has been reported that the charge transporting process is closely related to the trap characteristics of materials [30,31]. The thermally stimulated current method is an effective technique to study trap parameters as well as storage and transport characteristics of space charges in materials, and has been widely used in recent years [9,23,32,33]. In order to clarify the relationship between the transport process of surface charges and trap characteristics of pressboard samples, TSDC of oil-impregnated pressboard before and after modification were measured, and the results are shown in Figure 11.

From Figure 11, it can be clearly seen that the peak value of the NIP TSDC curve was 84 pA, while that of OIP was just around 52 pA. To make a quantitative analysis of the trap characteristics of NIP and OIP, trap parameters such as the quantity of trapped charges and trap energy level were also calculated according to the TSDC curves, and are summarized in Table 4.

In essence, traps are the localized states which can convert free charges into bound charges, and they can be divided into deep traps and shallow traps according to their energy level [32]. It is clear from Table 4 that both NIP and OIP possessed shallow traps, since the trap energy levels in NIP and OIP were 0.48 and 0.49 eV, respectively. Moreover, the quantity of trapped charges in NIP was 1.58 times greater than that in OIP, which suggested that the density of shallow traps in NIP was significantly increased by the modification with Fe_3_O_4_ nanoparticles, thus promoting the dissipation of space charges. For shallow traps, the potential barriers overcome by space charges during the jumping process are small, which can inhibit the accumulation of space charges in materials, thus leading to the decrease of space charge density [33]. 

From the above results, one conclusion that can be reasonably drawn is that, compared to that of OIP, the rapid surface charge decay rate in NIP is mainly attributable to its increased shallow traps density resulting from the addition of Fe_3_O_4_ nanoparticles, which is consistent with our previous studies [7,22]. In particular, the creeping flashover usually starts from the weakest link of oil-impregnated pressboard. The accumulation of space charges usually occurs at the interface of oil/pressboard due to the mismatches in the dielectric parameters of oil and pressboard when under an external electric field. For these reasons, the distortion of the electric field along the interface would be aggravated by these charges and the local electric field would increase dramatically, promoting the creeping flashover more easily. With the addition of Fe_3_O_4_ nanoparticles, plenty of shallow traps were produced in the NIP, which can promote the dissipation of surface charges, leading to the suppression of local electric field distortion caused by charge accumulation. Hence, the maximum intensity of the local electric field was effectively reduced, which contributed to improving the creeping flashover voltage of NIP under lightning impulse voltages.

## 4. Conclusions

This paper investigated the effects of Fe_3_O_4_ nanoparticles on the creeping flashover strength of oil-impregnated pressboard under lightning impulse stress, revealing the modification mechanism based on Fe_3_O_4_ nanoparticles in details. It can be convincingly concluded that, under applied voltages, NIP possesses higher resistance to creeping discharge than OIP at various electrode gap distances. 

The modification mechanism of the creeping flashover based on Fe_3_O_4_ nanoparticles is clarified as follows: with the addition of nanoparticles, a better match in relative permittivity between nanofluid and NIP would be obtained and the breakdown strength of the oil sample would be improved, thus promoting a more uniform and more substantial electric field distribution at the interface and improving the dielectric performance of nanofluid/pressboard composite insulation systems. Meanwhile, the density of shallow traps in NIP was also significantly increased due to the addition of Fe_3_O_4_ nanoparticles, which can promote the migration of surface charges and restrain the accumulation process, leading to the weakening of the distortion of the local electric field caused by charge accumulation and enhancement of the electric field distribution uniformity along the interface. Hence, the creeping flashover performance of oil-impregnated pressboard was apparently improved.

## Figures and Tables

**Table 1:** Chop time of OIP and NIP under positive lightning impulse voltages.

Electrode Gap Distance (mm)	OIP		NIP		Increase Rate
	AVG (μs)	SD (μs)	AVG (μs)	SD (μs)	(%)
20	12.5	1.5	21.6	0.7	72.8
30	17.3	1.0	28.8	0.7	66.5
40	22.1	1.4	36.0	1.5	62.9

**Table 2:** Chop time of OIP and NIP under negative lightning impulse voltages.

Electrode Gap Distance (mm)	OIP		NIP		Increase Rate
	AVG (μs)	SD (μs)	AVG (μs)	SD (μs)	(%)
20	25.0	2.8	27.1	4.6	8.4
30	30.1	4.6	30.3	5.6	0.6

**Table 3:** The relative permittivities of oil samples and pressboard samples.

Sample	Relative Permittivity		Permittivity Ratio of Oil to Oil-Impregnated Pressboard
	Oil (ε_oil_)	Oil-Impregnated Pressboard (ε_board_)	
OIP	2.2	4.2	0.52
NIP	3.0	4.4	0.68

**Table 4:** The trap parameters of NIP and OIP.

Samples	Quantity of Trapped Charges (nC)	Trap Energy Level (eV)
OIP	101.04	0.49
NIP	159.64	0.48
